# The Response of Ventral Tegmental Area Dopaminergic Neurons to Bupropion: Excitation or Inhibition?

**DOI:** 10.32598/bcn.9.10.250

**Published:** 2019-07-01

**Authors:** Shirin Sadighparvar, Fereshteh Tale, Parviz Shahabi, Somayyeh Naderi, Firouz Ghaderi Pakdel

**Affiliations:** 1. Neurophysiology Research Center, Urmia University of Medical Sciences, Urmia, Iran.; 2. Department of Physiology, School of Medicine, Urmia University of Medical Sciences, Urmia, Iran.; 3. Neuroscience Research Center, Tabriz University of Medical Sciences, Tabriz, Iran.; 4. Reproductive Health Research Center, Urmia University of Medical Sciences, Urmia, Iran.

**Keywords:** Bupropion, VTA, Dopaminergic neurons, Intraventricular injection, Iontophoresis

## Abstract

**Introduction:**

Antidepressants can modulate brain monoamines by acting on pre-synaptic and postsynaptic receptors. Autoreceptors can reduce the monoamines effect on the somatodendritic or pre-synaptic regions despite its postsynaptic counter effects. The direct effect of some antidepressants is related to its temporal and spatial bioavailability in the vicinity of these receptors (still a matter of controversies). This research evaluated the direct effect of acute bupropion on the Ventral Tegmental Area (VTA) dopaminergic neuronal firing rate.

**Methods:**

Male Wistar rats were divided into intracerebroventricular and microiontophoretic groups with 14 subgroups (n=5 in each subgroup). Amounts of 1, 0.5, 0.1, 0.01, 0.001, and 0.0001 mol of bupropion (5 μL/3 min) were microinfused to the first group and then the ejected amounts of bupropion at -500, -300, -150, -50 nA of electrical currents (1 mol, pH=4.5, 5 min) were applied to the second group. The control and sham subgroups were studied in each group, too. The units with stable firing rates were extracted, and the effect of bupropion was evaluated statistically with a P value less than 0.05 as the level of significance.

**Results:**

The highest amount of bupropion in the intracerebroventricular application could excite 42% of the neurons and inhibit 56% of them, but the highest amount of microiontophoretic application of bupropion could inhibit 97.5% of the neurons. The neuronal response to bupropion was dose-dependent in all treated groups.

**Conclusion:**

The dual effects of intracerebroventricular bupropion on the VTA dopaminergic neurons but solo inhibitory effect of its microiontophoretic application reflect the intra-VTA and extra-VTA heterogenic cellular and molecular control over the dopaminergic outflow that can be mediated by different receptors. The dopamine autoreceptors on the VTA dopaminergic neurons have complex modulatory effects on the dopaminergic response.

## Highlights

- Two routine methods of Intracerebroventricular and microiontophoresis applications are used to study the cellular effects of bupropion on the Ventral Tegmental Area (VTA) dopaminergic neurons.
- The dopaminergic VTA neuronal firings rates are extracted and assigned to evaluate the direct or indirect effects of bupropion.
- Bupropion can modulate the synaptic activity and post-synaptic neuronal spiking by the pre- or post-synaptic effects.
- Although the bupropion as an antidepressant, has excitatory effects on the dopaminergic neurons, its direct effect is inhibitory.
- The dissociation of direct and indirect effects of bupropion on the VTA dopaminergic neurons can explain some side effects of bupropion besides its anti-depressive property.

## Plain Language Summary

Antidepressants comprise the major part of prescribed drugs worldwide. Bupropion was introduced as an anti-depressant, but it was mainly used as smoke cessation. The mechanism of action of bupropion is not well known, and the wide range of its effects showed the complexity of its activities. This study examined the effects of indirect and direct application of bupropion on the cellular levels by intraventricular or iontophoretic methods, respectively. The findings of this study showed that the general indirect effect of bupropion on the dopaminergic VTA neurons is dual; excitatory, and inhibitory, but its direct effect is inhibitory. The dose-related presence of bupropion can show the different effects on the dopaminergic VTA neurons. These findings could explain some opposite effects and also side effects of bupropion.

## 1. Introduction

The monoamine hypothesis of depression is a crucial pharmacologic issue for depression based on tricyclic antidepressants and monoamine oxidase inhibitors effects. These drugs mimic serotonin, norepinephrine, and or dopamine. These property has directed research studies to evaluate the role of biogenic monoamine neurotransmitters in depression (Schildkraut, 1967). Dopamine insufficiencies and or deficiencies associated with depression along with endocrine disorders (Diehl & Gershon, 1992). There is some clinical evidence on the role of midbrain dopamine, Ventral Tegmental Area (VTA) in depression (Brown & Gershon, 1993), but it could also be involved in motivation, addiction, and psychosis. Increase in the dopamine in the VTA by Monoamine Oxidase Inhibitors (MAOIs) and or dopamine reuptake inhibitors provide well-known pharmacologic agents to alleviate depression (Randrup & Braestrup, 1977; Sampson, Willner, & Muscat, 1991; Willner, 1983).

The VTA comprises of dopamine, Gamma-Aminobutyric Acid (GABAergic), and glutamate-releasing neurons, most of which are dopaminergic and GABAergic (Holly & Miczek, 2016; Nair-Roberts et al., 2008; Yamaguchi, Wang, Li, Ng, & Morales, 2011). Two primary afferents of the VTA; mesolimbic and mesocortical systems, originate from nucleus accumbens, amygdala, hippocampus, prefrontal cortex, and limbic system (Albanese & Minciacchi, 1983; Fallon, 1988; Le Moal & Simon, 1991). The efferents of the VTA send back to the nucleus accumbens to produce modulatory loop (Spanagel, Herz, & Shippenberg, 1992). Enhancing the synaptic dopamine availability increases the postsynaptic responses is associated with depression pharmacotherapy, and for this reason, the agents with elevated dopamine and or other monoamines have profound antidepression outcomes.

Reuptake inhibition of the multi-neurotransmitters has provided a novel and effective generation of antidepressants, such as bupropion (Dremencov et al., 2004; Dremencov et al., 2005; Nutt et al., 2006). Bupropion (Wellbutrin) that is recently re-marketed in the USA and other regions exerts its antidepressant effect by blocking the reuptake of dopamine. Bupropion has a weak inhibitory effect on norepinephrine reuptake, too (Horst & Preskorn, 1998). The confirmed anti-smoke effects of bupropion are believed to antagonize the nicotinic acetylcholine receptors (nAchRs) (Taeron, 2002). Although it is reported that bupropion, primarily blocks the reuptake of both dopamine and norepinephrine, the mechanism of its action has not been fully elucidated (Ascher et al., 1995). The dual effect of bupropion on the dopamine and norepinephrine reuptakes has changed the treatment of specific symptoms of depression, cognitive, and fatigue associated with selective monoamine inhibitors and selective serotonin reuptake inhibitors (SSRIs) (Fabre, Brodie, Garver, & Zung,1983; Green, 1997). Bupropion also has less risk of sexual dysfunction compared to SSRIs (Pereira, Arias-Carrion, Machado, Nardi, & Silva, 2014).

Although bupropion primarily inhibits the dopamine reuptake, its precise mechanism of action is still unknown because of differences in its in vivo and in vitro effects. The in vitro potency of norepinephrine reuptake inhibition of bupropion is about twice than that in vivo (Ascher et al., 1995).

The duration of its administration has different effects on norepinephrine-releasing neurons. In acute systemic application, it can reduce norepinephrine releasing firing rates, but its sustained administration can recover the baseline firing rate (Ghanbari, El Mansari, & Blier, 2010). This effect is suggested due to the somatodendritic α2-adrenoceptors activation (Dong & Blier, 2001). Besides its action on monoamines, a low amount of bupropion can attenuate nicotine-evoked dopamine release in striatum in slice preparations (Mansvelder, Fagen, Chang, Mitchum, & McGehee, 2007; Sidhpura, Redfern, Rowley, Heal, & Wonnacott, 2007). Nicotine can induce significantly large somatic responses in the neurons (Klink, de Kerchove d’Exaerde, Zoli, & Changeux, 2001; Wooltorton, Pidoplichko, Broide, & Dani, 2003) via alpha7-nicotinic receptors and modulate nicotine-induced reinforcement and extracellular dopamine in the mesolimbic system (Besson et al., 2012). The mechanism of the bupropion action on the VTA dopaminergic neurons is not well-known due to the presence of different dopamine receptors and their various locations and destinations pre- and postsynaptically.

Our recent studies showed that acute systemic infusion of bupropion could decrease formalin-induced pain behavior in rats (Naderi, Pakdel, Osalou, & Cankurt, 2014). Microinfusion of the bupropion into the locus coeruleus nuclease could decrease formalin-induced pain response (Jahanbani et al., 2014). The intracerebroventricular application of the bupropion revealed that it could reduce the spontaneous firing of locus coeruleus neurons dose-dependently (Pakdel et al., 2014). The effect of the bupropion on the dendritic adrenergic receptors has been postulated as a critical factor for these actions. It is reported that neither 2 nor 14 days of bupropion (30 mg/kg/d) administration alter the firing and burst activity of dopamine neurons (El Mansari, Ghanbari, Janssen, & Blier, 2008), but there are other reports about increase in firing rate with 14 days administration of bupropion at two 10 and 20 mg/kg/d doses (West & Weiss, 2011). Cooper et al. revealed that the acute systemic infusion of bupropion could inhibit the firing of the VTA dopaminergic neurons (Cooper et al., 1994).

Bupropion can also act on the VTA dopaminergic neurons by VTA GABAergic neurons. The microinfusion of the bupropion could inhibit the neuronal firing of the putative VTA GABAergic neurons dose-dependently (Amirabadi et al., 2014). We hypothesized that the critical neuronal control of the VTA dopaminergic neurons is the GABA neurotransmission and because there is no evidence about the direct effect of bupropion on VTA dopaminergic neurons, this study was designed to test the acute effects of bupropion via intracerebroventricular and microiontophoretic applications on the dopaminergic neuronal firing rates of the VTA.

## 2. Methods

### 2.1. Ethical approval

The committee for biomedical research ethics approval reviewed the procedure, experiments, protocols, and all guidelines for the care and use of experimental animals. The Animal Laboratory Center of Urmia University of Medical Sciences (ALCUUMS) supervised precisely over all ethical issues. All experimental procedures and protocols were approved by the Urmia Medical Science Research Ethics Committee (UMSREC). The ethical issues were performed in accordance with the National Institute of Health (NIH) guide for the care and use of laboratory animals.

### 2.2. Study animals

Healthy male Wistar rats of 250–280 g weight (Pasteur Institute, Tehran, Iran) were housed (three on a cage) at a 12/12 h light/dark cycle (7:00 AM to 7:00 PM). The ambient temperature was controlled (22±2°C), and the food and water were at labium. The two intracerebroventricular and microiontophoretic categories of animals were divided into 14 groups (5 per each subgroup) according to the bupropion application with the sham and control groups too. Apart from the control and sham groups, in the first category, the animals received 1, 0.5, 0.1, 0.01, 0.001, and 0.0001 mol of intracerebroventricular bupropion (5 μL/3 min). In the second category, bupropion was ejected by -50, -150, -300, and -500 nA electrical currents (1 mol of bupropion solution, pH=4.5, 5min) with the control and sham subgroups. Briefly, animals were anesthetized by urethane (1.2 g/kg) initially, and the booster dose (∼ 15% to 25% of the initial dose) was used when there were any discomfort signs. The core body temperature was continuously monitored and maintained at ∼37°C during the entire experiment. The stereotaxic coordinations of bregma and interaural landmarks were determined for each rat, and the location of recording and injecting electrodes were exposed according to the Paxinos and Watson stereotaxic rat brain atlas (Paxinos & Watson, 2007).

### 2.3. VTA multiple electrophysiological recording

Multiunit extracellular electrophysiological activity of the VTA dopaminergic neurons was recorded as described previously (Mejías-Aponte & Kiyatkin, 2012). The animals were mounted into a stereotaxic frame (Steolting, USA) and recording electrodes were directed via a burr hole to the core portion of the VTA on the bregma zero-zero plane. The recording barrel of glass micropipette (A-M systems, USA) was filled with 2.0% of Pontamine Sky Blue solution in 0.5% sodium acetate and lowered into the brain to the VTA (Bregma=–6.84, ML=±0.5, and DV=8.6 mm from the bregma zero-zero plane). Microiontophoretic ejection was carried out by three barrel micropipettes; recording, microinjecting, and countercurrent micropipettes. Microelectrode impedance was checked (Sutter Instruments BV-10M, USA, in vitro impedance at 1000 Hz, about 5±2 MΩ) and adjusted to recording and or microiontophoretic application.

Multiunit signals were amplified (10000×) with a high impedance digital amplifier (Electromodule 3111, ScienceBeam, Tehran, Iran), filtered (bandpass 300–3000 Hz), and acquisition (sample rate=50000) by the high-speed USB port on a PC running Windows 7.0 Premium Pro. Only spontaneously active right VTA dopaminergic neurons were included in the data sheets. The electrophysiological criteria have been explained for isolation of putative VTA dopaminergic neurons in previous articles (Marinelli, Cooper, Baker, & White, 2003; Mejias-Aponte & Kiyatkin, 2012; White & Wang, 1984). Spontaneous low discharge rate (<10 spikes/second), long-duration of spikes (>2.5 ms), and a triphasic (+/–/+) or biphasic with a notch in the positive component were considered as the main criteria.

After stable firing, the baseline firing for 10 min was carried out and continued until the effect disappeared. In the control and sham groups, recordings continued about 120 min. The PSTH (peri-stimulus time histogram) of units was calculated at 1 ms bin size. Online PSTH analysis used for detection of firing pattern change. The PSTH of all recordings was calculated by using Igor Pro 6.0 (Wavemetrics, USA) software offline. The PCA (Principle Component Analysis) signal processing protocol with duration, amplitude, rising and falling slope criteria (±5.0% accuracy) was used for PSTH calculation. All units with no pattern changing and stable spontaneous firing were included. The obtained data in all sessions were expressed as Mean±SD.

### 2.4. Bupropion application

In intracerebroventricular groups, a 30-gauge stainless steel injection needle was connected to a 10-μL Hamilton syringe (Hamilton Bonaduz AG, Switzerland) with a PE-10 polyethylene tube (A-M systems, USA) used for microinjection. The injection pipette was lowered to the ipsilateral cerebral ventricle core, and the recording electrode was located into the right VTA. The drug vehicle (fresh artificial cerebrospinal fluid or ACSF) and different concentrations of bupropion were injected at 11^th^ to 13^th^ min with a programmable motorized microsyringe pump (New Era Pump Systems Inc., NY, USA). In the sham groups, the drug vehicle (fresh ACSF) was microinfused at the same time, and recording continued up to 120 min. In groups with intracerebroventricular microinjection, the response of 322 neurons were extracted as control (n=44), sham (n=41), and n=38, n=40, n=41, n=39, n=41, and n=38 for 1, 0.5. 0.1, 0.01, 0.001, and 0.0001 mol of bupropion, respectively.

In the second groups, microiontophoretic application of the bupropion was carried out with a microiontophoretic system (WPI 260-B, USA). Briefly, 3-barrel micropipettes (A-M Systems, USA) were pulled (David Kopf puller, 700C DKI, USA) with adequate stem and shank. One micropipette was filled with normal saline to eject a counter current, the second with recording solution as described previously, and the last one with bupropion solution (1 mol, pH=4.5). The bupropion solution was prepared by dissolving the bupropion into the sterile distilled water, and the pH of the solution was adjusted by using the 1 mol HCl and 1 mol NaOH solution. Before microiontophoretic application of the bupropion, a holding current (+20 nA) was passed through the injection micropipette, and an equal countercurrent (–20 nA) was ejected via countercurrent micropipette to cancel current effects. The ejecting currents at -50, -150, -300, and -500 nA with balanced countercurrents were used for bupropion ejection. In microiontophoretic groups, the response of 229 neurons as n=43, n=41, n=39, n=40 for -500, -300, -150, and -50 nA analysed respectively in addition to the control (n=32) and sham (n=34) groups.

### 2.5. Data analysis

The Kolmogorov-Smirnov test (K-S test) was used as a goodness of fit-test for the statistical probability distribution of data for parametric or non-parametric statistical tests. As mentioned previously, the single units were isolated with Igor Pro 6.0 under the PCA protocol. The PSTH was calculated off-line for all recordings. The average of the pre-microinfusion period (the first to the tenth minute) was used for each recording as a pre-stimulus period, and its firing rate was used as a baseline PSTH. The data in the post-injection period were analyzed point-to-point to determine the statistically significant difference. One-way repeated measures ANOVA with Tukey’s post hoc test was used to evaluate the statistical difference. The Igor Pro 6.0 software was used for statistical data analysis with P<0.05 as the least acceptable level of significance.

### 2.6. Histological verification

Pontamine Sky Blue dye was deposited at the end of the experiments for making the recording location with the passing of −20 μA electrical current through the recording electrode (∼10 min). The animals were deeply anesthetized in the final session, perfused transcardially with 10% phosphate-buffered formalin solution, and their brains were removed and fixed in the perfused solution. Coronal sections (40 μm) were taken by a microtome (SLEE, London). The trajectory path and the location of the micropipette tips and infusion cannula were observed under a light microscope for verification. The misinjected rats were excluded from the analysis.

### 2.7. Drugs, chemicals, and instruments

The sources of drugs, chemicals, and instruments were as follows. Bupropion, formalin, Pontamine Sky Blue, fast cresyl violet, urethane were purchased from Sigma-Aldrich (Sigma-Aldrich, USA). Sodium acetate and sodium chloride were purchased from Merck (Merck Darmstadt, Germany). Polyethylene microtube (A-M systems, USA) and Hamilton micro-syringes (Hamilton Bonaduz AG, Switzerland), motorized microsyringe pump (New Era Pump Systems Inc., NY, USA), microiontophoretic system (WPI 260-B, USA), micropipettes (A-M Systems, USA), pipette puller (David Kopf, 700C DKI, USA), impedance check instrument (Sutter Instruments BV-10M, USA) were prepared from standard sources.

## 3. Results

The data mining based on PCA and analyzing of the PSTH of neuronal firing were used for inter- and intra-group statistical analysis. The PSTH recording for 10 min before microinfusion or microiontophoresis was analyzed as a pre-stimulus time histogram. In the sham and bupropion-treated groups, the microinjection of the vehicle (fresh ACSF or quasi-saline solution, respectively) or drug solution was carried out at 11^th^ to 13^th^ min of the recording. Injection of drug vehicle in sham groups showed no significant difference between pre-injection and post-injection.

Briefly, the data of 485 neurons with spontaneous activity were isolated as putative VTA dopaminergic neurons and presented. Isolated 322 and 229 neurons in groups with the intracerebroventricular and microiontophoretic application of bupropion respectively were included. In the intracerebroventricular application of bupropion, 135 (42%) neurons got excited, 180 (56%) of neurons got inhibited, and 7 (2%) of them showed no response. The maximum stimulatory effects began 59.3±8.1 s post-injection on average and lasted 35.1±5.5 min, but the maximum inhibition began 46.2±7.5 s post-injection on average and lasted 42.2±08.2 min. In the microiontophoretic application of bupropion, 159 (97.5%) neurons got inhibited, and only 2 (2.5%) of them slightly got excited, but 1 neuron showed no response. The maximum inhibition lasted 51.7±18.2 min on average.

### 3.1. The shape of VTA dopaminergic extracellular spikes

**Figure 1.**
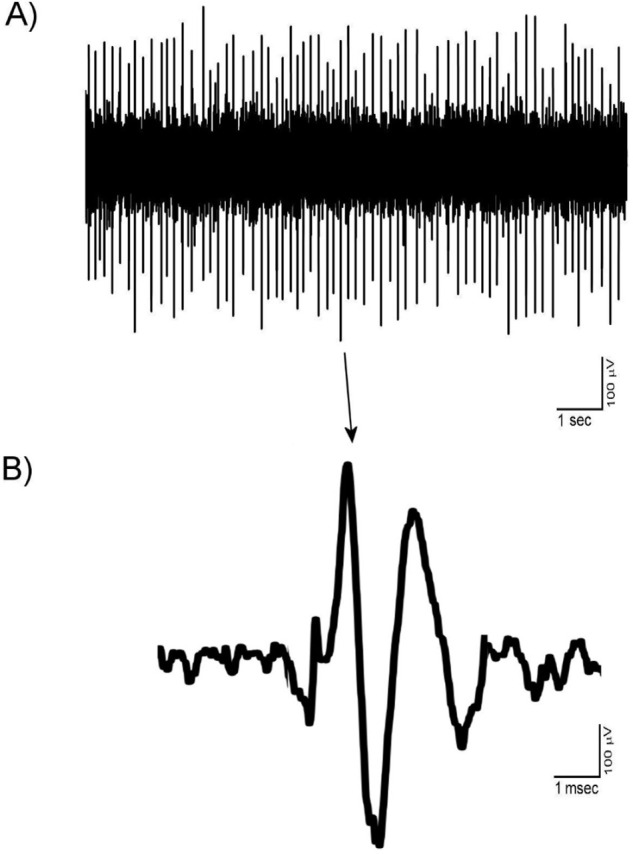
A typical multiunit recording of the VTA dopaminergic neurons in the control group A. A trace of the multiunit recording; B. A typical spike signature of VTA dopaminergic neuron isolated from the mentioned multiunit recording. The VTA dopaminergic neurons usually have a triphasic extracellular spike shape with the firing rate less than 10 spikes/second. The presented neuron had three phasic (+/−/+) shape with a notch at the beginning of the first + phase. The mean amplitude of the presented neuron was about 700 μV peak-to-peak. VTA dopaminergic neurons showed a low spontaneous firing rate (<10 spikes/second), and the Mean±SD presented sample rate was 4.13±0.82 spikes/second.

The mentioned criteria for isolation of the VTA dopaminergic neurons as the main elements of the PCA protocol used in the Igor software. The units isolated in the control groups showed the stability and reproducibility of the recordings. All isolated recordings had no sensitization-desensitization properties and pattern change. Figure 1 shows a trace (A) and a typical spike signature (B) a recording from VTA dopaminergic units. The Mean±SD peak-to-peak amplitude of the spikes was 510±46 mV. The VTA dopaminergic neurons showed a low spontaneous firing rate (<10 spikes/second).

### 3.2. VTA dopaminergic neuronal activity in the control and sham groups

**Figure 2.**
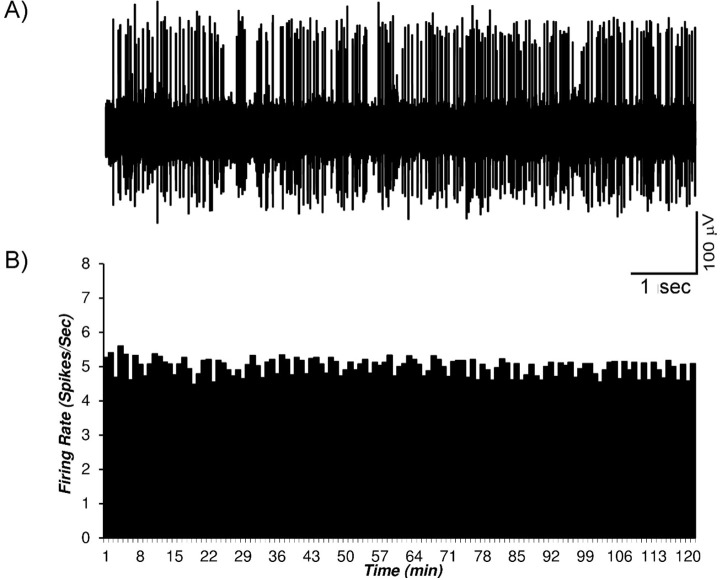
A sample of the multiunit firing of the VTA dopaminergic neurons in the control group A sample trace of the multiunit firing of the VTA dopaminergic neurons in the control group; B. The histogram of the firing of the VTA dopaminergic neurons of the control group. In the control group with naive animals, the VTA dopaminergic neuronal firing under standard condition was recorded up to 120 min. The Mean±SD firing rate of the neurons in the control group was 4.97±1.2 spikes/second. The mean firing in each minute calculated from the averaged firing in each second in every minute. The data are presented as Mean±SD.

The control group animals (naive animals) under standard condition were anesthetized by urethane and the VTA dopaminergic neurons recorded. The neuronal firing was recorded up to 2 hours for the stability of the recordings. The mean firing in each minute was calculated from the averaged firing in each second in every minute. Figure 2 shows firing of a trace (A) of a multi-unit recording and the histogram (B) of the mean VTA dopaminergic neuronal firing in the control group. The mean firing rate of the selected neuron was 4.97±1.2 spikes/second during 120 min.

**Figure 3.**
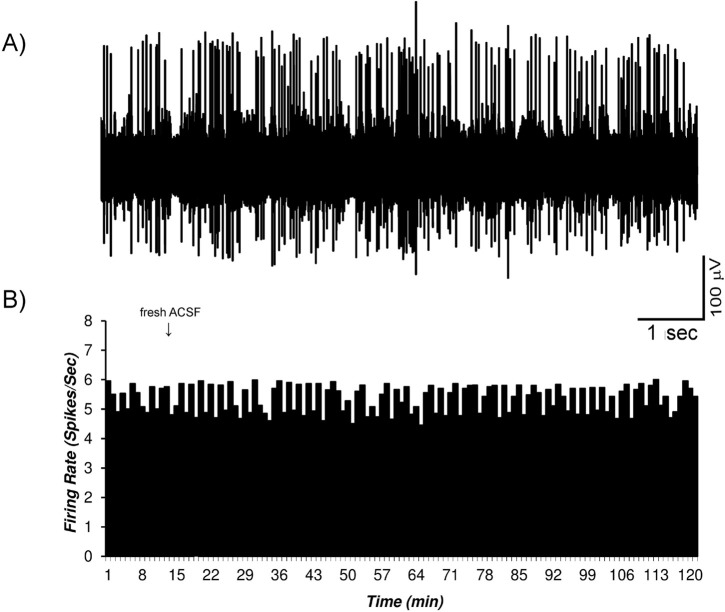
The firing of the VTA dopaminergic neurons in the sham group with the intracerebroventricular application of the bupropion A. The firing of sample VTA dopaminergic multiunit neurons in the sham group. In the sham group, the VTA dopaminergic neuronal firings under standard condition were recorded up to 120 min. Bupropion vehicle (sterile ACSF, 5 μL/3min, in 11^th^ to 13^th^ min) was injected by 30-gauge Hamilton syringe in the core of right ventricle with motorized programmable pump; B. The histogram of the neuronal firing rate in the recorded neurons. The Mean±SD neuronal firing rate was 5.34±0.5 spikes/second. Vehicle had no significant effect on the neuronal firing. The paired student t-test was used for statistical analysis. The data are presented as Mean±SD.

The sham groups in this study were intracerebroventricular sham group with microinjection of fresh ACSF and microiontophoretic application of the balanced solution at pH=4.5 that used for bupropion microiontophoretic application. Figure 3 shows the use of the fresh ACSF into the right ventricle of a sampled recording of the VTA dopaminergic neurons. The Mean±SD neuronal firing rate in the sham groups was 5.34±0.5 spikes/second. Vehicle had no significant effect on the neuronal firing. The statistical paired student t-test was used for statistical analysis.

**Figure 4.**
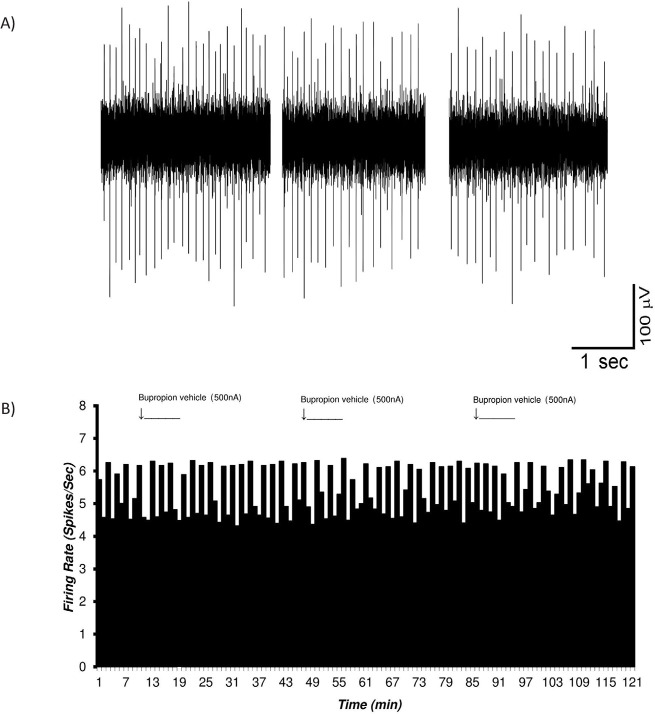
The firing of the VTA dopaminergic neurons in the sham group with the microiontophoretic application A. A multiunit firing trace of a sample of VTA dopaminergic neurons in the sham group in the presence of microiontophoresis current. In the sham group, the VTA dopaminergic neuronal firing under standard condition was recorded up to 120 min. The ejecting current (500 nA, 1 mol, pH=4.5, 5 min) was applied in the ejecting micropipette 3 times in the recording. The Mean±SD firing rate in this neuron was 4.72±0.5 spikes/second, and the ejecting current with balanced counter current injected. One-way repeated measures ANOVA was used for PSTH data analysis, and there was no significant effect on the firing of the pre- and post-ejecting periods. The data are presented as Mean±SD. B. The histogram of the neuronal firing rate in the recorded neurons.

In the microiontophoretic groups, the adjusted solution (1 mol, at pH=4.5) and ejected amounts to±500 nA of electrical current for 5 min were used in the sham group. Figure 4 shows a sample trace of the sham group VTA dopaminergic neuronal firing (A) and the histogram of the mean firing rate of the neurons in the sham group (B). The Mean±SD firing rate of the VTA dopaminergic neurons was 4.72±0.5 spikes/second. The microiontophoretic injecting of the bupropion vehicle was repeated 3 times to evaluate the precise vehicle and current effects. The ejecting current with balanced countercurrent was injected. Two-way repeated measures ANOVA was used for PSTH data, and there was no significant effect on the firing.

### 3.3. Neuronal activity of neurons following intracerebroventricular application of bupropion

The intracerebroventricular application of the bupropion showed dual, stimulatory, and inhibitory responses in the VTA dopaminergic neurons. Bupropion in the highest injected amount could excite 42% and inhibit 56% of neurons, but 2% of them showed no response. The maximum stimulatory effects began 59.3±8.1 s after intracerebroventricular infusion on average and lasted 52 min, but the maximum inhibition occurred 46.2±7.5 s after it on average and continued 78 min. The responses of the neurons were dose-dependent.

#### 3.3.1 Stimulatory effect of the intracerebroventricular application of the bupropion

**Figure 5.**
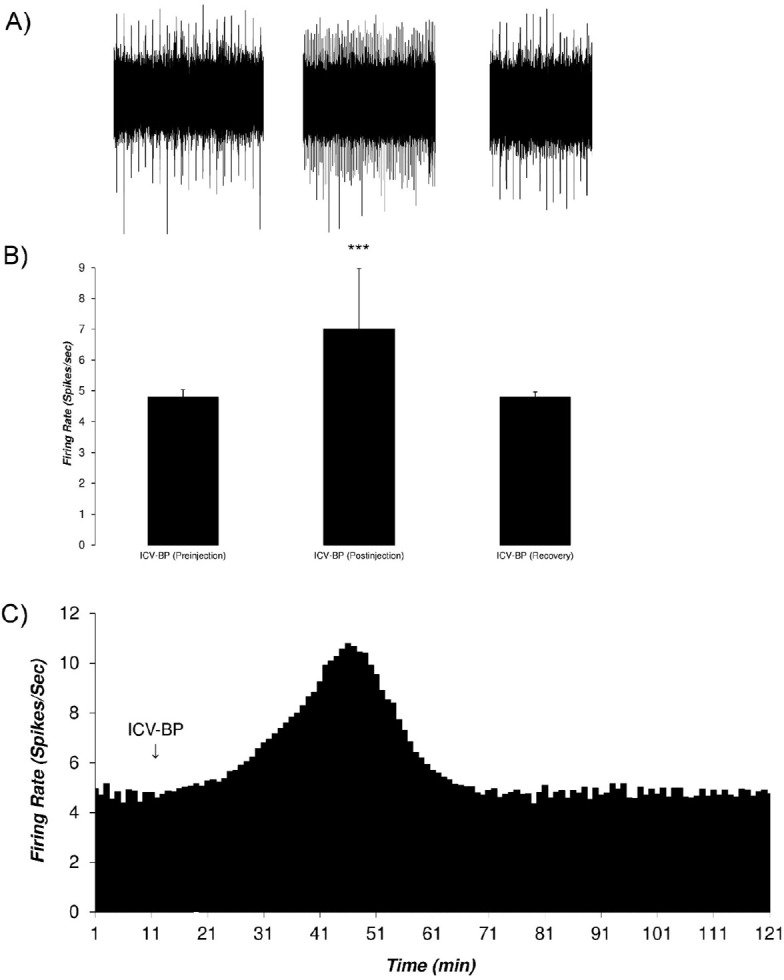
The figure of the VTA dopaminergic neurons to intracerebroventricular application of the bupropion with excitatory effects A. A sample of the multiunit recording shows the recorded trace in three phases of pre-injection, post-injection, and recovery after microinjection of bupropion (1 mol, 5 μL/3min, at 11^th^ to 13^th^ min) into the ipsilateral ventricle. The average firing rate before and after consecutive minutes for each minute was calculated for determining the statistical difference for each minute of the isolated unit; B. The bar graph of the average firing rate of the isolated VTA dopaminergic neuron in each period. The excitatory periods with the highest firing rate has a statistical difference with pre-injection and recovery periods. Bupropion elicited a profound excitation in the neuron; C. Continuous histogram of mean firing rate in three mentioned phases. The Mean±SD firing rate of the pre-injection period was 4.8±0.24 spikes/second. The post-injection period was included in a period with excitation of the neuron, that began at 12^th^ to 68^th^ min of the recording. In the excitation period, the firing rate increased to 7.01±1.96 spikes/second with a maximum of firing 10.8 spikes/second in the 33^rd^ min. The recovery period was determined by firing that came back to its pre-injection value.The mean firing rate of the excitation period showed a statistically significant difference. One-Way repeated measures Analysis of Variance (ANOVA) with the Tukey’s post hoc test were used for statistical evaluation. The data are represented as Mean±SD with a significant level of ^***^ P<0.001; ICV-BP=Intracerebroventricular application of bupropion.

Figure 5 shows typical neuronal firing in response to the intracerebroventricular administration of the bupropion (1 mol, 5 μL/3 min, at 11^th^ to 13^th^ min). The PSTH of the pre-injection, post-injection, and the recovery period are also showed. In this Figure, the neuron was stimulated up to 66 min. The average±SD neuronal firing rate in the pre-injection period, was 4.8±0.24 spikes/second. The intracerebroventricular application of bupropion could excite the neuron with the Mean±SD firing rate of 7.01±1.96 spikes/second with the maximum of 10.8 spikes/second in the 33^rd^ min after injection. In the recovery period, the neuronal firing returned to its baseline firing at 4.8±0.17 spikes/second. One-way repeated measures ANOVA with the Tukey’s post hoc test revealed that the firing rate had a significant difference (P<0.001).

**Figure 6.**
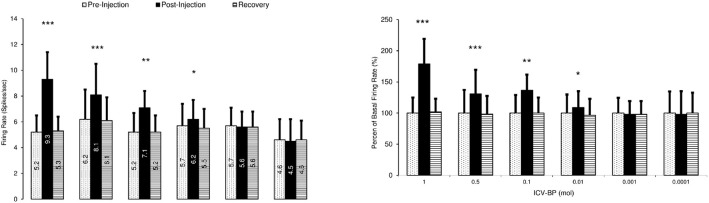
Bar graph of the excitation response of the VTA dopaminergic neurons to the intracerebroventricular application of bupropion in different groups The upper graph shows the mean of firing rate of the VTA dopaminergic neurons per different amount of bupropion (1, 0.5, 0.1, 0.01, 0.001, and 0.0001 mol, 5 μl/3min, at 11^th^ to 13^th^ min) and the lower graph shows the percentage of firing rate according to the pre-injection firing as 100%. Bupropion could excite the neurons dose-dependently. There were significant differences between the mean firing rates of the excitation periods of 1, 0.5, 0.1, and 0.01 mol of bupropion but the amounts of 0.001 and 0.0001 mol did not show any significant difference. One-way repeated measures ANOVA with Tukey’s post hoc test were used for statistical evaluation. The data are represented as Man±SD with significant levels of ^***^ P<0.001; ^**^ P<0.01; and ^*^ P<0.05; ICV-BP= Intracerebroventricular application of bupropion.

Figure 6 shows the VTA dopaminergic neuronal firing rates of groups in response to different amounts of intracerebroventricular bupropion administration. The Figure shows that the amounts of 1, 0.5, 0.1, 0.01 mol of bupropion had significant stimulatory effects on the neurons, but 0.001 and 0.0001 mol of bupropion had no significant impact. These data revealed that intracerebroventricular application of the bupropion could excite 42% of neurons at the highest amount. The maximum firing intensity of excited neurons was 119% of the baseline firing. One-way repeated measures ANOVA with the Tukey’s post hoc test revealed that the firing rate had a significant difference (P<0.001) in the excitatory period compared with pre-injection and recovery periods. The Figure also shows that the stimulatory effect of bupropion is dose-dependent.

#### 3.3.2 Inhibitory Effect of the intracerebroventricular application of the bupropion

**Figure 7.**
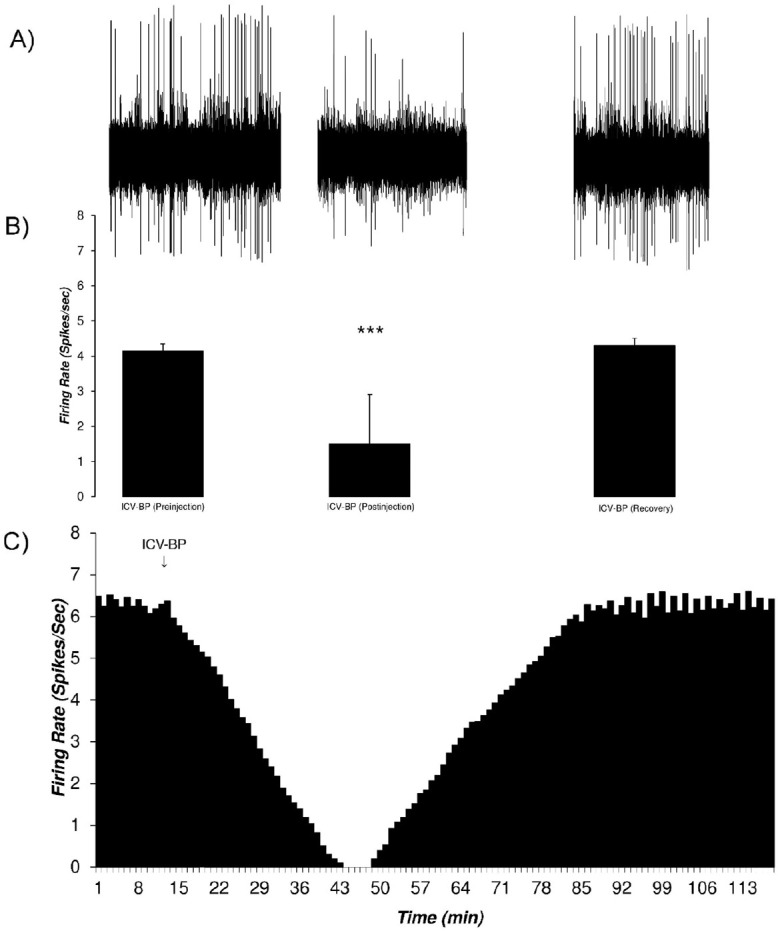
The response of the VTA dopaminergic neurons to intracerebroventricular application of the bupropion with inhibitory effects. A. A sample of the multiunit recording shows the recorded trace in three phases of pre-injection, post-injection, and recovery before and after microinjection of bupropion (1 mol, 5 μL/3min, at 11^th^ to 13^th^ min) into the ipsilateral ventricle. The average firing rates of the before and after consecutive minutes for each minute was calculated for determining the statistical difference for each minute of the isolated unit; B. The bar graph of the average firing rate of the VTA dopaminergic neuron in each period. The inhibitory periods with the least firing rate has a statistical difference with pre-injection and recovery periods. Bupropion elicited a profound inhibition in the neuron; C. Continuous histogram of mean firing rate in three mentioned phases. The Mean±SD firing rate of the pre-injection period was 6.3±0.14 spikes/second. The post-injection period was included in a period with inhibition of the neuron that began at 14th to 68th min of the recording. In the inhibition period, the, Mean±SD firing rate decreased to 3±1.94 spikes/second with a minimum of 0 spikes/second firing in the 44^th^ to 48^th^ min of recording. The recovery period was the period that the firing rate returned to its pre-injection period (6.27±0.19 spikes/second). The middle bar graph shows the comparison of the neuronal firing in the pre-injection, inhibition, and recovery periods. The mean firing rate of the inhibition period showed a statistically significant difference. One-way repeated measures ANOVA with the Tukey’s post hoc test were used for statistical evaluation. The data are presented as Mean±SD with significant level of ^***^ P<0.001; ICV-BP=Intracerebroventricular application of bupropion.

Figure 7 shows the typical inhibitory response of the VTA dopaminergic neuron to the intracerebroventricular application of the bupropion (1 mol, 5 μL/3 min, at 11^th^ to 15^th^ min). In this Figure, the firing of the neurons decreased up to 71 min. The average±SD neuronal firing rate in the pre-injection period was 6.32±0.14 spikes/second. The intracerebroventricular microinjection of the bupropion (5 μL/3 min) inhibited the neurons. The average±SD firing of the neurons in the inhibitory period was 3.00±1.95 spikes/second with a minimum of 0 spikes/second during 44^th^ to 48^th^ min after the injection. In the recovery period, the neuronal firing returned to the baseline firing with 6.3±0.19 spikes/second on average. One-way repeated measures ANOVA with the Tukey’s post hoc test revealed that the firing rate had a significant difference (P<0.001).

**Figure 8.**
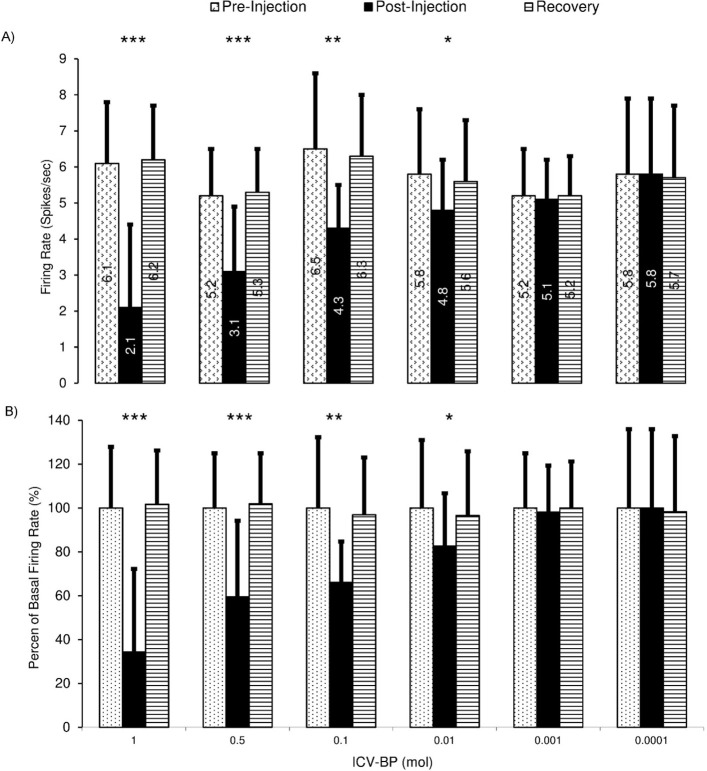
Bar graph of the inhibition effect of bupropion on the VTA-DA neurons in different groups A. The graph shows the mean firing rate of the ventral tegmental area dopaminergic neurons to different amount of bupropion application (intracerebroventricular, 1, 0.5, 0.1, 0.01, 0.001, and 0.0001 mol, 5 μL/3min, at 11^th^ to 13^th^ min) and lower graph; B. Shows the percentages of firing rate according to the pre-injection firing as 100%. Bupropion inhibited the neurons dose-dependently. There were significant differences in the mean firing rate of the inhibition period of 1, 0.5, 0.1, and 0.01 mol of bupropion but the amounts of 0.001 and 0.0001 mol did not show any significant difference. One-way repeated measures ANOVA with Tukey’s Post Hoc test were used for statistical evaluation. The data are represented as Mean±SD with significant level of ^***^ P<0.001; ^**^ P<0.01; ^*^ P<0.05; ICV-BP=Intracerebroventricular application of bupropion.

Figure 8 shows the effect of intracerebroventricular microinjection of bupropion on the neuronal firing rates in the bupropion-treated groups. The Figure shows that bupropion in the amounts of 1, 0.5, 0.1, 0.01 mol had significant inhibitory effects on the neurons, but 0.001 mol of bupropion had no effect. These data revealed that intracerebroventricular microinjection of bupropion could inhibit 56% of neurons at the highest amount. The minimum firing intensity was 0% of its baseline. One-way repeated measures ANOVA with the Tukey’s post hoc test revealed that the firing rate had a significant difference (P<0.001). The Figure also shows that the stimulatory effect of bupropion is dose-dependent.

### 3.4. Neuronal activity of VTA dopaminergic neurons following microiontophoretic application of the bupropion

Microiontophoretic application of the bupropion in the VTA could change the dopaminergic neuronal activity. The electrical currents of -50, -150, -300, and -500 nA were applied to the bupropion solution (1 mol, pH=4.5, 5 min). In these groups, the highest ejected amount of bupropion could inhibit 97.5% of neurons, 1.8% of them had slight non-significant excitation while 0.7% (1 neuron) of neurons showed no response. In this dose, the maximum inhibition lasted 96 min with a silent period of about 30 min.

#### 3.4.1 Inhibitory effect of microiontophoretic application of bupropion on the VTA dopaminergic neuronal activity

**Figure 9.**
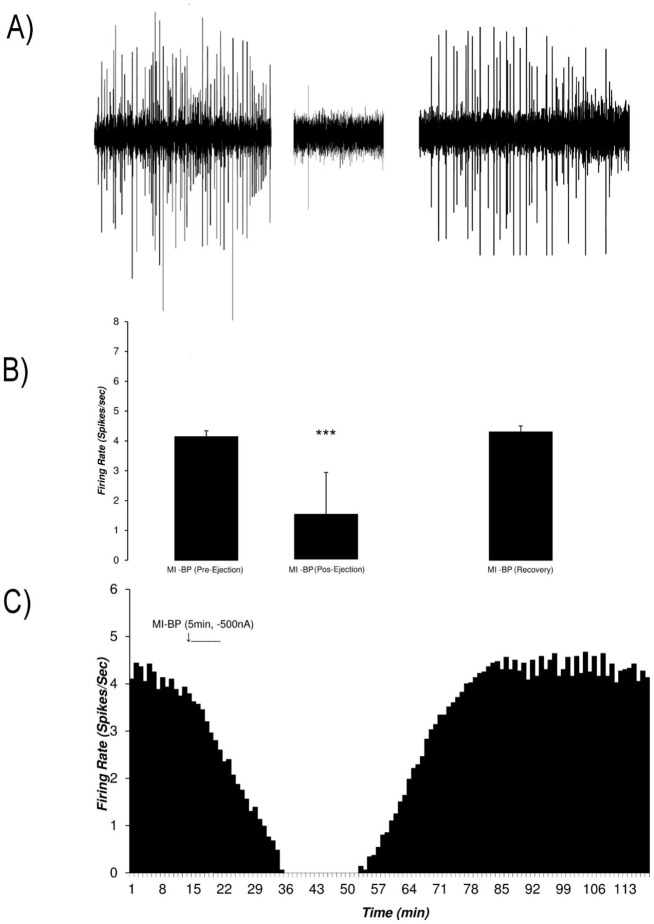
The response of VTA dopaminergic neurons to microiontophoretic application of the bupropion A. A sample of the multiunit recording shows the recorded trace in three phases of pre-injection, post-injection, and recovery before and after microiontophoretic application of bupropion (-500 nA, 1 mol, pH=4.5, 5 min, at 11^th^ to 15^th^ min). The average firing rate of the before and after consecutive minutes for each minute was calculated for determining the statistical difference for each minute of the isolated unit; B. The bar graph of the average firing rate of the VTA dopaminergic neuron in each period. The inhibitory periods with the least firing rate has a statistical difference with pre-injection and recovery periods. Bupropion elicited a profound inhibition in the neuron; C. Continuous histogram of mean firing rate in three mentioned phases. The Mean±SD firing of the pre-ejection period was 4.14±0.2 spikes/second. The post-ejection period is included in a period with inhibition of the neuron that began at 11^th^ to 75^th^ min of the recording. In the inhibition period, the firing rate decreased to 1.5±1.4 spikes/second with a minimum of 0 spikes/second firing in the 35^th^ to 52^nd^ min of recording. The recovery period was the period that the firing rate returned to its pre-injection period (4.3±0.2 spikes/second). The middle histogram shows the comparison of the neuronal firing in the pre-ejection, inhibition, and recovery periods. The mean firing rate of the inhibition period showed a statistically significant difference. One-way repeated measures ANOVA with the Tukey’s Post Hoc test were used for statistical evaluation. The data are represented as Mean±SD with the significant levels of ^***^ P<0.001; MI-BP= Microiontophoretic application of bupropion.

Microiontophoretic application of the bupropion revealed the direct effect of the bupropion on the neurons. This effect showed mainly inhibitory nature. Figure 9 shows typical neuronal inhibition by microiontophoretic application of the bupropion (-500 nA, 1 mol, pH=4.5, 5 min). The average±SD firing rate in pre-ejection period was 4.14±0.2 spikes/second. Microiontophoretic application of the bupropion inhibited the neuron with the Mean±SD firing rate of 1.5±1.4 spikes/second in the inhibitory period. The neuron was silent for 17 min during 36^th^ to 52^th^ min of recording. In the recovery period, the Mean±SD neuronal firing returned to baseline with 4.3±0.2 spikes/second. The neuron inhibited about 63 min on average. One-way repeated measures ANOVA with the Tukey’s post hoc test revealed that the firing rate had a significant difference (P<0.001).

**Figure 10.**
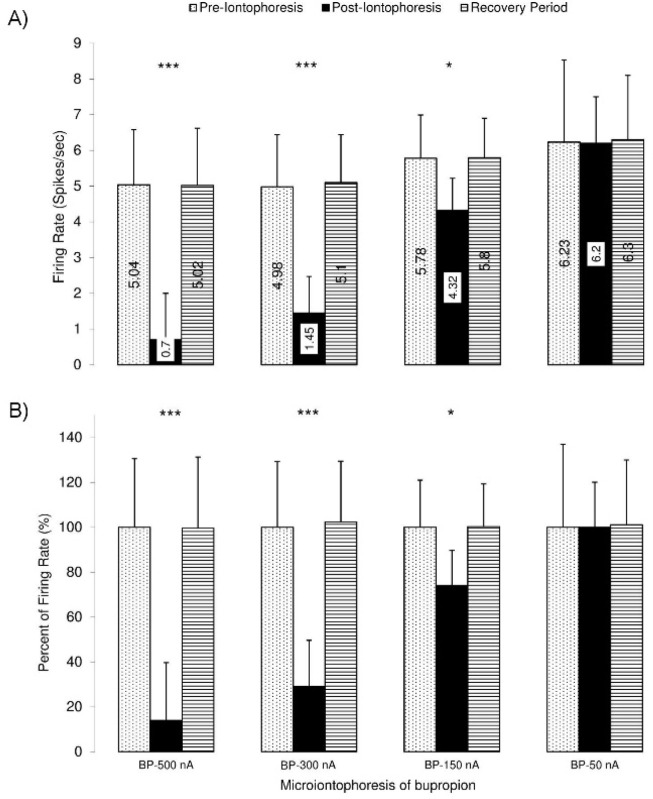
Bar graph of the inhibition of the VTA dopaminergic neurons to microiontophoretic application of the bupropion in different amounts A. The upper graph shows the mean firing rate of the VTA dopaminergic neurons at different amounts of bupropion application (-500, -300, -150, and -50 nA, 1 mol, pH=4.5, at 11^th^ to 15^th^ min); B. The lower graph shows the percentage of the firing rate according to the pre-injection firing as 100%. Bupropion inhibited the neurons dose-dependently. There were significant differences between the mean firing rate of the inhibition period of -500, -300, and -150 nA of bupropion but the amount of -50 nA did not show any significant difference. One-way repeated measures ANOVA and the Tukey’s Post Hoc test were used for statistical evaluation. The data are represented as Mean±SD with significant levels of ^***^ P<0.001; ^**^ P<0.01; ^*^ P<0.05.

Figure 10 shows the microiontophoretic application of the bupropion with different amounts on the VTA dopaminergic neuronal firing rates. The Figure shows that the ejected amounts of -500, -300, -150 nA of bupropion could inhibit the neurons significantly. The -50 nA of bupropion had no significant effect. These data revealed that microiontophoretic application of the bupropion could inhibit 97.5% of neurons at the highest ejected amount. The minimum firing rate of the inhibited neurons was 0% of the baseline firing. The mean firing rate was 13.9%, 29.1%, 74.1% at -500, -300, and -150 nA of bupropion, respectively but at -50 nA it was 101.1% of baseline firing. One-way repeated measures ANOVA with the Tukey’s post hoc test revealed that the firing rate had a significant difference (P<0.001). The Figure also shows that the inhibitory effect of bupropion is dependent to the ejected amount.

**Figure 11.**
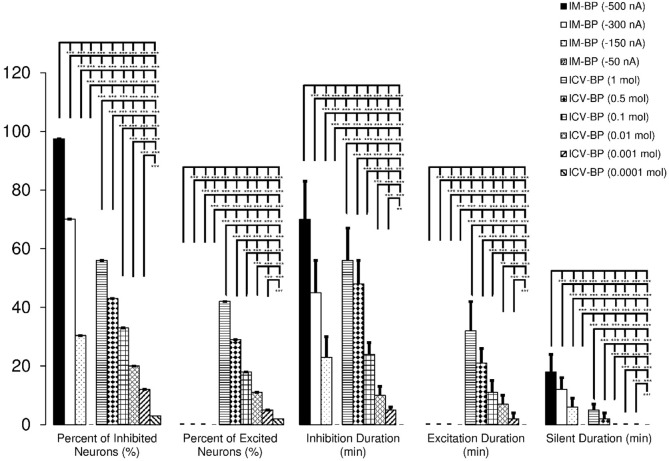
Cumulative data that present the direct inhibition of the Bupropion (BP) on the VTA dopaminergic neurons The effect of Microiontophoretic (MI) and Intracerebroventricular (ICV) application of the bupropion on the percentage of inhibited neurons, percentage of excited neurons, inhibition duration, excitation duration, and silent duration of the VTA dopaminergic neurons in different groups. Bupropion inhibited 97.5%, 70.1%, and 30.4% of VTA dopaminergic neurons at -500, -300, -150 nA, respectively without response at -50 nA. The percentages of inhibited neurons in ICV application of bupropion were 56%, 43%, 33%, 20%, 12%, and 3% at amounts of 1, 0.5, 0.1, 0.01, 0.001, and 0.0001 mol, respectively. The percentage of excited neurons in MI application of bupropion was 0% but they were 42%, 29%, 18%, 11%, 5%, and 2 % at 1, 0.5, 0.1, 0.01, 0.001, and 0.0001 mol in ICV application of bupropion, respectively. The Mean±SD duration periods of inhibition were 70±13, 45±11, 23±7 min at -500, -300, -150 nA, respectively without any inhibition at -50 nA. The Mean±SD duration periods of inhibition of neurons in the ICV application of bupropion were 56±11, 48±8, 24±4, 10±3, 5±1 min at the amounts of 1, 0.5, 0.1, 0.01, 0.001 mol, respectively without inhibition at 0.0001 mol. The mean excitation duration in MI application of bupropion was 0, but in the ICV application of bupropion, they were 32±10, 21±5, 11±4, 7±3, 2±2 min at amounts of 1, 0.5, 0.1, 0.01, 0.001 mol, respectively without excitation at 0.0001 mol. The MI application of bupropion made VTA dopaminergic neurons silent for 18±8, 12±4, 6±3 min at -500, -300, -150 nA, respectively without any silent neurons at -50 nA. The ICV application of bupropion made VTA dopaminergic neurons silent for 5±2, and 2±2 at 1 and 0.5 mol, respectively. However, there were no silent neurons at the remaining amounts. The crosstab statistical test was used for statistical evaluation of descriptive data (percentage of inhibited and excited neurons). One-way repeated measures ANOVA and Tukey’s post hoc test were used for quantitating data evaluation. The data are presented as Mean±SD with significant levels of ^***^ P<0.001; ^**^ P<0.01.

Table 1 summarizes the firing rate of the VTA dopaminergic neurons in response to intracerebroventricular and microiontophoretic administration of the bupropion. The data indicate no significant differences between the inhibitory effects of bupropion, and the direct effect of bupropion lacks stimulatory effects. Figure 11 shows the percentages of inhibited and excited neurons, inhibition and excitation duration, and the silent duration of the VTA dopaminergic neurons. The Figure shows the dose-dependent and direct inhibitory effect of bupropion.

**Table 1.** The VTA dopaminergic neuronal response to the intracerebroventricular and microiontophoretic application of bupropion

**VTA Dopaminergic Neuronal Response**	**Microiontophoretic Application (nA)**				**Intracerebroventricular Application (mol)**					

	**-500**	**-300**	**-150**	**-50**	**1**	**0.5**	**0.1**	**0.01**	**0.001**	**0.0001**
% of inhibited neurons	97.5	70.1	30.4	0	56	43	33	20	12	3
% of Excited Neurons	0	0	0	0	42	29	18	11	5	2
Inhibition duration (min)	70±13	45±11	23±7	0	56±11	48±8	24±4	10±3	5±1	0
Excitation duration (min)	0	0	0	0	32±10	21±5	11±4	7±3	2±1	0
Silent Duration (min)	18±6	12±4	6±3	0	5±2	2±2	0	0	0	0

ICV. Intracerebroventricular; MI: Microiontophoresis; nA. nano Ampere; mol. mole; VTA-DA. Ventral Tegmental Area Dopaminergic; Min. Minute

**Figure 12.**
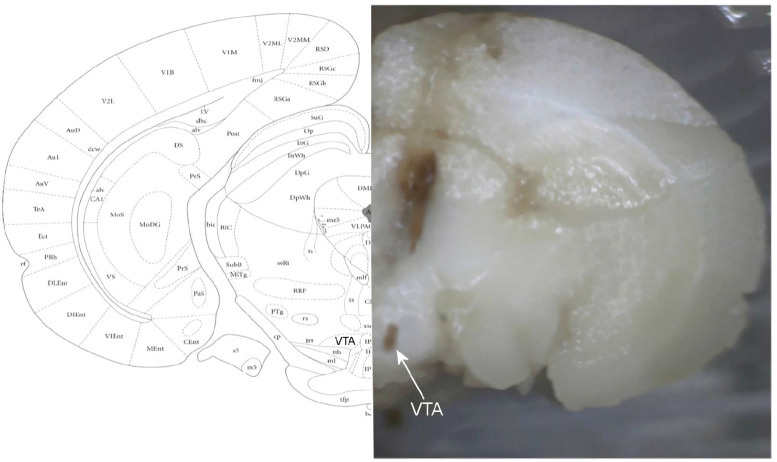
A photomicrograph of the coronal section indicates the location of the trajectory to and micropipette tip location in the right VTA In the final session, the implanted rat on the stereotaxic frame received −20 μA of electrical current for 10 min through the recording electrode to deposit microiontophoretic of Pontamine Sky Blue in the recording site. The whole brain was precisely removed and maintained in the 10% phosphate-buffered formalin solution for at least 1 week. The brains were cut by using a microtome to evaluate the location of electrode tips. In the Figure, the location of the VTA is indicated on the histologic image (right) and stereotaxic coordination (left).

As mentioned in the “Materials and Methods” section, in the final session of the experiments, the Pontamine Sky Blue was deposited in the tip location of the micropipettes by passing −20 μA of electrical currents. The brains were removed precisely after transcardial perfusion by 10% phosphate-buffered formalin solution until completion of the brain fixation. The brains were sectioned coronally to take 40-μm thick slices by a microtome. The trajectory path and location of the micropipette tips in addition to the infusion cannulae loci were evaluated. Figure 12 shows a sample of a photomicrograph on the right side of the fixed brain. In the Figure, the location of dye deposition is shown on the right side, and stereotaxic boundary of the VTA is shown on the left side.

## 4. Discussion

This study shows that acute intracerebroventricular microinjection of bupropion has dual stimulatory and inhibitory effects on putative VTA dopaminergic neurons, but the direct microiontophoretic application of the bupropion predominantly indicates inhibitory effect. The distribution of the bupropion all over the brain can affect differently VTA dopaminergic neurons directly and or indirectly. The results of this article provide the unique perspective of the direct effect of the bupropion using the microiontophoretic technique.

Depression prevalence is increasing over the past decades. Developing effective pharmaceutical agents to treat and prevent depression calls for an understanding of the etiology and mechanism of depression. The second generation of antidepressants has provided successful drugs with low side effects. The VTA and some of its targets are central to the initiation and development of drug dependence and psychiatric disorder, i.e. depression. The VTA dopaminergic neurons show different responses to antidepressant agents according to time and approach of application. This behavior reflects the existence of hidden mechanisms in the controlling of their responses. The regulation process of these neurons is complicated due to heterogeneity and differences in their pharmacological properties (Kalivas, 1993). Despite some clinical evidence suggesting the bupropion as a weak dopamine reuptake inhibitor, its mechanism of action is not well-known yet (Dong & Blier, 2001).

### 4.1. The VTA heterogeneity, core play roles of intra-VTA microcircuits

The different brain regions have special internal circuitry to transmit proper signals associated with their afferent nature. Immunohistochemical staining for tyrosine hydroxylase revealed diverse subcellular populations of the VTA. Dopaminergic and GABAergic populations are the first and second groups, and others are tertiary ones. This main classification has been demonstrated by electrophysiological and pharmacological evidence (Cameron, Wessendorf, & Williams, 1997). The co-release of glutamate in the axon terminals of some VTA-dopaminergic efferents in the nucleus accumbens results in further heterogeneity of the VTA (Hnasko, Hjelmstad, Fields, & Edwards, 2012; Stuber, Hnasko, Britt, Edwards, & Bonci, 2010).

Recently, the homogeneity of the VTA-dopaminergic neurons is under question due to functional differences. The most common in vivo electrophysiological studies of the VTA dopaminergic neurons propose these neurons as a single homogenous population. The simple heterogeneity of the VTA dopaminergic neurons based on electrophysiological properties is expressed on the long spike duration (>2.5 ms) and low firing rate (<10 spikes/second). The D2-agonist-induced hyperpolarization and or presence of large Ih currents due to cyclic nucleotide-dependent hyperpolarization-activated cation channels are the other tools for distinguishing putative VTA dopaminergic from non-dopaminergic neurons (Ungless & Grace, 2012). However, there are controversial findings that show the overlap of these criteria between two classes of VTA neurons (Lammel et al., 2008; Zhang, Placzek, & Dani, 2010). The diversity of putative VTA dopaminergic neurons may reflect the presence of differences in the responses of dopaminergic efferents of the VTA and the diversity of dopaminergic functional properties.

### 4.2. Dopamine receptors: Pre-synaptic and post-synaptic locations with D1-like and D2-like diversity

The brain dopamine receptors have different profiles concerning their location and classification. Pharmacological and genetic tools have determined multiple subtypes of the D1-like and D2-like receptors. Two major families of D1-like receptors are the D1, D5, and three major families of D2-like receptors are D2, D3, and D4 receptors. D1-like and D2-like receptors show opposite intracellular effects on the cAMP production, i.e. increasing and decreasing effects, respectively. D1-like receptors activate phospholipase C, and D2-like receptors activate K channels and inhibit calcium channels (Spano, Govoni, & Trabucchi, 1978). The modulatory effects of the dopamine receptors on the neuron can produce a cascade of protein kinase A and after-activation of terminal signaling proteins (Beaulieu & Gainetdinov, 2011). The density of the D1-like and D2-like dopamine receptors are different in the brain regions. D1-like receptors are the dominant form of dopamine receptors in the postsynaptic non-dopaminergic neurons. High expression of D1-like receptors was found in the frontal cortex, substantia nigra, striatum, olfactory bulb, and amygdala. D2-like receptors are predominantly found in the dopaminergic as well as in non-dopaminergic neurons postsynaptically. However, in dopaminergic neurons, the massive D2-like receptors are found in the pre-synaptic membrane and somatodendritic loci. D2-receptors with average densities are located in the substantia nigra, ventral tegmental area, hypothalamus, septum, amygdala, hippocampus, and the cortical regions (Beaulieu & Gainetdinov, 2011; Moritz, Free, & Sibley, 2018).

D1-like dopamine receptors are rare in the VTA dopaminergic neurons, and D2-like receptors have important roles. The presence of the D1-like receptors in the VTA non-dopaminergic neurons is predominate, and their agonist can create the stimulatory effect on VTA dopaminergic neurons indirectly.

### 4.3. Stimulatory effects of bupropion on the VTA dopaminergic neurons: The prominent in-direct effect

The stimulatory effects of an antidepressant may arise from the monoamine hypothesis of depression and its pharmacotherapy. The first idea about the mechanism of the stimulatory effects of bupropion on the VTA dopaminergic neurons is based on the overall impact of antidepressants. The stimulatory effects of bupropion may reflect the presence of the stimulatory receptors on the VTA dopaminergic neurons for dopamine because bupropion inhibition of dopamine reuptake can increase dopamine availability to produce excitation. The direct dopamine stimulatory effects on the VTA dopaminergic neurons may be produced by D1-like receptors on the VTA dopaminergic neurons while there is no substantial evidence to support this hypothesis. The density of D1-like receptors on the VTA dopaminergic neurons is very low compared with the density of D2-like receptors.

Direct stimulatory effects of bupropion on the VTA dopaminergic neurons can be due to the action of the dopamine transporter to induce excitation because of its coupling to chloride ions (Horn, 1990). This activity, as seen in the amphetamine fast ionotropic excitatory response to depolarization, is caused by the inhibition of the chloride influx. The fast in vitro amphetamine-induced currents is comparable with glutamatergic ionotropic currents (DeFelice & Goswami, 2007).

The indirect stimulation of the VTA dopaminergic neurons is the most acceptable hypothesis. The GABAergic input to the VTA dopaminergic neurons is one of the major inhibitory inputs, and bupropion alone can reduce the inhibitory GABAergic input to dopaminergic neurons. The VTA dopaminergic neurons are under the tonic discharge of GABAergic inputs from medium spiny neurons of the nucleus accumbens. These neurons exert a tonic inhibitory effect on the VTA dopaminergic neurons and suppress their activity (Haber, Wolfe, & Groenewegen, 1990; Kalivas, Churchill, & Klitenick, 1993; Nauta, Smith, Faull, & Domesick, 1978). The nucleus accumbens has a noticeable effect on the neuronal activity of VTA and VTA-nucleus accumbens interaction has a key role in the firing rate (Moaddab, Kermani, Azizi, & Haghparast, 2013).

On the other hand, bupropion as a nicotinic acetylcholine receptors (nAChRs) antagonist can inhibit nicotine effects on GABA neurons, and the nAChRs blockade can remove the excitatory drive or disinhibition to GABA neurons (Fryer & Lukas, 1999; Slememer, Martin, & Damaj, 2000). The GABAergic innervation blockade enhances neuronal activity with a disinhibition mechanism (Johnson & North, 1992). The nAChRs in the VTA neurons can also contribute to their nicotine rewarding effects (Nisell, Nomikos, & Svensson, 1994).

The excitation of VTA dopaminergic neurons by acute micro-infusion of bupropion is consistent with the emerging views on the dopamine role in reward-based learning and memory processes (Berridge, 2007; De Biasi & Dani, 2011; Schultz, 2007). Increase in neuronal activity of VTA can amplify and synchronize the output of DA signaling to different targets such as the nucleus accumbens. Recent studies show that VTA neuronal activity enhances rewards and correlates with the increase in synchronization of neuronal spiking (Cryan, Bruijnzeel, Skjei, & Markou, 2003; De Biasi & Dani, 2011; Joshua et al., 2009).

Inhibition of the dopamine reuptake can increase the synaptic dopamine content. The onset of bupropion excitatory effect on the neurons has many delays in comparison with its inhibitory effects. This difference indicates that excitation could be generated by afferent neurons, not by their somatodendritic receptors. Increase in discharge rate by bupropion can also be explained by a decrease in tonic inhibitory inputs to the VTA. In this study, the changes in neuronal activity, excitation, and inhibition began 60±10.5 s and 8±4.4 s respectively after the maximum amount of bupropion. This delay in the onset of bupropion effects suggests that its excitatory effects on the neurons are carried out via indirect effect or probable disinhibition of GABAergic inputs to the VTA. We propose that different time courses from inactivation of nAChR subtypes on GABA inputs to the neurons can contribute to the bupropion-delayed excitatory effect. The distinct contributions of different nAChRs in VTA dopaminergic neurons have been already defined in many studies. In addition to nicotine, activation of α4α6β2* nAChRs in the neurons is sufficient to support the initiation of cellular changes. α4α6β2* nAChRs may be a promising target for future drug addiction (Engle, Shih, McIntosh, & Drenan, 2013). The postulated differences in distinct contributions of α4* and α6* nAChRs in the soma vs. the axon terminal of nucleus accumbens neurons are related to neuronal firing properties (Exley et al., 2011).

### 4.4. Inhibitory effects of bupropion on the VTA dopaminergic neurons: The prominent direct effect

Based on our study results, the inhibitory effects of the microiontophoretic application of bupropion were much stronger than its excitatory effects on VTA dopaminergic neuronal activity. Several possible mechanisms can cause inhibitory effects.

The direct inhibitory effects of the bupropion on VTA dopaminergic neurons are due to the occupation of the D2-like dopamine receptors. The major D2-like dopamine receptors are autoreceptors on the somatodendritic regions and strongly inhibit the VTA-dopaminergic neurons by lowering intracellular cAMP. Lejeune and Millan (1995) reported that the systemic application of the D3 dopamine receptor selective agonists could inhibit VTA dopaminergic neuronal firing rate that is blocked by D3 dopamine receptor antagonists’ dose-dependently both of them (Lejeune & Millan, 1995). The activation of the D2 dopamine receptors on the VTA dopaminergic neurons in the wild-type mice could produce profound hyperpolarization, but in the D2–/– mice, it was abolished due to receptor lacking. However, the subtype of the D2 dopamine receptor is essential because of its response to activation. On the other hand, the overall results show that the D2 dopamine autoreceptors on the VTA dopaminergic neurons have important hyperpolarizing effects (Centonze et al., 2002). By inhibiting the reuptake of the dopamine, bupropion stimulates the condition of the D2 autoreceptors and hyperpolarize the VTA dopaminergic neurons.

A second idea about direct inhibitory effects of bupropion on the VTA dopaminergic neurons, which is considered recently, is the role of dopamine transporter to induce inhibition. This protein with high presynaptic density is a sodium ion- and chloride ion-dependent (Horn, 1990). Its activation by drugs such as amphetamine produces profound hyperpolarization because of inhibiting the sodium influx by cotransport with dopamine. Ionotropic outcomes of dopamine transporter inhibition make it a candidate for the authentic ionotropic-like receptor. The fast in vitro amphetamine-induced currents is comparable with GABAergic ionotropic current. The dopamine transporter in this state couples with chloride shift to enhance the hyperpolarization (DeFelice & Goswami, 2007). The inhibition of the dopamine transporter by increasing the dopamine post-application of bupropion may prevent the influx of sodium that results in hyperpolarization of the VTA-dopaminergic neurons.

The antagonistic effect of the bupropion on nAChRs is the idea based on smoke cessation of the bupropion. The nAChRs are present in the neurons and participate in their excitation and inhibition (Mansvelder, Keath, & McGehee, 2002). The presynaptic nAChRs in the VTA have a functional association with glutamatergic inputs to the VTA and can enhance LTP occurrence in the neurons. Presynaptic excitatory potentials of nAChRs indicate that bupropion can also inhibit the neurons from presynaptic nAChRs antagonism (Mansvelder & McGehee, 2000).

There is evidence of the antagonistic effect of bupropion on α3β2* and α3β4* nAChRs respectively in rats’ striatum and hippocampus across the same concentration range that inhibits dopamine and norepinephrine transporter functions (Miller, Sumithran, & Dwoskin, 2002). The same effect of bupropion can contribute to its inhibitory effect on the neurons. Nicotine or its agonists on nAChRs activation can excite the neurons to release dopamine. nAChR knockout mice express a modified response to nicotine application. The subunits of nAChRs are a key part of the response to nicotine or its antagonist. Activation of β2*-nAChR switches the neurons from a resting state to an excited state (Mameli-Engvall et al., 2006). The study of different nAChRs on neuronal activity reveals that direct governing stimulation and or disinhibition can excite or inhibit the neurons and endogenous acetylcholine in them (Graupner, Maex, & Gutkin, 2013). Bupropion can antagonize and inhibit nAChRs on the neurons with direct and fast inhibition responses.

The precise response of the neurons to the route of the drug application is vital for its design and time of the delivery. Researchers have recently focused on the pharmacological responses of the agents on the reward system because of their wide spectrum use and abuse in the world. The VTA dopaminergic neurons that can produce a massive and functional dopaminergic out-flow of the brain are the core part of this focus. However, in contrast to some dopaminergic nuclei, about 55% of neurons in the VTA are dopaminergic and putatively selected neurons can reveal the heterogeneity of VTA neuronal response. The high density of D2 dopamine receptors on the somatodendritic region of the VTA dopaminergic neurons has a critical functional modulatory effect on these neurons. Evidence of inhibitory and excitatory effects of bupropion with its direct local application reveal that temporal and spatial availability is essential for its nature of effect.

In summary, this study presents new data on bupropion effects on the VTA dopaminergic neuronal firing rate, whereas many previous studies have administered bupropion systemically and only explained its general action. Our obtained results emphasize that bupropion can excite or inhibit the neurons, while its direct effect on them is inhibitory.
